# Effect of linguistic framing and information provision on attitudes towards induced seismicity and seismicity regulation

**DOI:** 10.1038/s41598-022-15448-4

**Published:** 2022-07-04

**Authors:** Darrick Evensen, Adam Varley, Lorraine Whitmarsh, Patrick Devine-Wright, Jen Dickie, Phil Bartie, Hazel Napier, Ilaria Mosca, Colin Foad, Stacia Ryder

**Affiliations:** 1 Politics and International Relations, University of Edinburgh, 22 George Square, Edinburgh, EH8 9LF Scotland, UK; 2 Biological and Environmental Sciences, University of Stirling, Stirling, UK; 3 Psychology, University of Bath, Bath, UK; 4 Geography, University of Exeter, Exeter, UK; 5 Mathematical and Computer Sciences, Heriot-Watt University, Edinburgh, UK; 6 British Geological Survey, Nottingham, UK; 7 British Geological Survey, Edinburgh, UK; 8 Politics and International Relations, University of Edinburgh, Edinburgh, UK

**Keywords:** Psychology and behaviour, Energy policy, Political economy of energy

## Abstract

Shale gas is an expanding energy source worldwide, yet ‘fracking’ remains controversial. Amongst public concerns is induced seismicity (tremors). The UK had the most stringent induced seismicity regulations in the world, prior to instating a moratorium on shale gas development. The Government cited induced seismicity as the key rationale for its November 2019 English moratorium. Yet, little is known about how the public perceives induced seismicity, whether they support regulatory change, or how framing and information provision affect perceptions. Across three waves of a longitudinal experimental UK survey (N = 2777; 1858; 1439), we tested whether framing of induced seismicity influences support for changing regulations. The surveys compared (1) quantitative versus qualitative framings, (2) information provision about regulatory limits in other countries and (3) seismicity from other industries, and (4) framing a seismic event as an ‘earthquake’ or something else. We find low support for changing current policy, and that framing and information provision made little difference to this. The one strong influence on perceptions of seismic events came from the type of activity causing the event; shale gas extraction clearly led to the most negative reactions. We discuss implications for future UK policy on shale gas and geothermal energy in an evolving energy landscape.

## Introduction

Regulation of induced seismicity—small earth tremors caused by human activity—became a make-or-break issue for the shale gas industry in the United Kingdom in 2019. The directors of both INEOS and Cuadrilla—two of the companies most involved in shale gas exploration—stated that the then-allowed magnitude for seismicity caused by hydraulic fracturing in 2019 was too low to allow exploration and production to move forward^1^. Under former Prime Minister Theresa May’s Conservative Government, the UK repeatedly stated it had no intentions to review the regulations, which required all fracturing operations to cease for eighteen hours if a seismic event at 0.5 M_L_ (Richter local magnitude) or greater occurred. This led to continued industry pressure and the resignation of the Shale Gas Commissioner for the UK, Natascha Engel, in April 2019, who claimed that ‘laws designed to prevent earthquakes and tremors are tantamount to a ban on fracking’^2^. The CEO of INEOS described the seismic regulations as ‘archaic’ and ‘unworkable’^3^. In an open letter to *The Times* in February 2019, several UK geoscientists advocated an increase from 0.5 to 1.5 M_L_ as the level that triggers the cessation of hydraulic fracturing operations^4^.

The ‘tantamount’ ban on shale gas extraction became an official political moratorium in November 2019 (for England that is—political moratoria existed already in Scotland, Wales, and Northern Ireland). Although this moratorium arguably was created to diffuse a thorny political issue for the Conservatives in advance of a general election, the predominant rationale the Government gave for it was the induced seismicity of August 2019, which saw a series of tremors as high as 2.9 M_L_^5^. Since the magnitude scale is logarithmic, each whole number increase in magnitude represents a tenfold increase in measured amplitude, and about 32 times the energy released.

A (tectonic or induced) event of 0.5 M_L_ will only be detected by sensitive monitoring equipment near the epicentre of an earthquake. Although earthquakes of 2.5 M_L_ or less are usually not felt, but are recorded by a seismograph, they can be rarely felt depending on the local conditions where the epicentres occur^6–8^. Thresholds for halting hydraulic fracturing operations in other jurisdictions, such as California (USA) and British Columbia (Canada), are set at 2.7 M_L_ and 4.0 M_L_, respectively^9^. These magnitudes correspond to an earthquake 158 and 3,162 times ‘bigger’ (i.e., in terms of earthquake size), and 1,995 and 177,828 times ‘stronger’ (i.e., in terms of seismic energy release) than a 0.5 M_L_ event^10^. From a public perception standpoint, however, this does not necessarily matter. The UK is not California nor British Columbia in terms of shale gas perceptions^11,12^. There are many reasons perceptions could differ, not least the ‘anchoring and adjustment heuristic’, which explains that once an initial plausible value for something is established, that creates a heavy psychological anchor than can only be dragged to a new location in cases of substantial motivation to seek a new value^13^. The UK Government having in place a seismicity threshold for seven years (2012–2019) conceivably provided a strong rationale for this being a valid value, and one can imagine little motivation on behalf of the general public to seek to re-anchor their beliefs in this respect.

The 2019 moratorium on shale gas extraction, alongside the UK hosting of the UNFCCC’s COP26 in November 2021 and the associated political rhetoric about ‘net-zero’ and ‘low-carbon’, seemed to suggest that discussion on shale gas would not return to mainstream UK politics^14^. Nevertheless, Russia’s invasion of Ukraine and the economic sanctions resulting from the war have led to renewed conversations about energy security globally, including in the UK. Whilst Prime Minister Boris Johnson’s current comments relate primarily to growth in renewables and facilitating expansion of North Sea oil and gas^15^, Cuadrilla CEO Francis Egan has unsurprisingly branded the conflict as a rationale to urgently ‘lift the shale gas moratorium and use these and additional wells to produce domestic shale gas’^16^. On 5 April 2022, Kwasi Kwarteng MP, Secretary of the UK Department for Business, Energy and Industrial Strategy (UK BEIS), commissioned the British Geological Survey to review the scientific evidence on shale gas extraction—with a report due in June 2022—to inform whether any change in the moratorium or seismicity regulations is warranted^17^.

The strong, and continually increasing, opposition to shale gas extraction in the UK cautions against the belief that hydraulic fracturing would return even now^14,18^; nonetheless, the very conversation about its revival did not even seem plausible in February 2022. We must note that throughout this article we use the terms ‘shale gas extraction’ and ‘fracking’ interchangeably. This is in line with how the terms are used in public discourse^19–21^; in colloquial use, these terms typically refer to the full range of processes and outcomes associated with shale gas/oil exploration, extraction, processing, transport, and development via hydraulic fracturing. In this article, however, we are predominantly concerned with aspects of shale gas extraction that could induce seismicity.

Aside from any implications for shale gas extraction, understanding public perceptions of induced seismicity is important due to the expanding role seen by Government and industry for deep geothermal energy in the coming decades^22^. In March 2022, a tremor of 1.7 M_L_^23^ was recorded in association with ‘testing operations’ at a deep geothermal well in Cornwall, England^24^, resulting in an operational pause for 24 hours for monitoring. The British Geological Survey estimates subsurface heat resources are sufficient to deliver 100 years of heat supply for the entire UK, and to provide 85% of Scotland’s and 9% of England’s electricity, with no intermittency^25,26^.

In addition to seismicity from deep geothermal well drilling^27,28^, induced seismicity in the UK could arise from development of compressed air storage, carbon capture and storage, and subsurface hydrogen storage. Globally, additional important causes of induced seismicity include: reservoir impoundment, sub-surface fluid removal, wastewater injection, erecting tall buildings, excavation of tunnels, nuclear explosions, coal mining, enhanced oil recovery, and carbon sequestration^29–32^. In our research, we focused on the causes of induced seismicity most relevant to and recognisable by the UK public.

Because political decision making is often concerned with and responds, at least in part, to public sentiment on national energy policy^33–35^, our research explored what the UK public thinks about induced seismicity from hydraulic fracturing and why they think this. We examined whether additional information about the tremors would influence their perspectives on the issue. In the third wave of our longitudinal survey, we expanded the focus on shale gas to include other causes of seismicity: deep geothermal operations, quarry blasting, and natural tectonic movements.

## Literature review

Literature on social acceptance of energy production refers to interdependences between socio-political, market, and community acceptance^36,37^; here, we stress the wider policy significance of societal perceptions on the under-researched topic of induced seismicity from hydraulic fracturing. The role of induced seismicity in leading to, or at least publicly justifying, the English moratorium on shale gas extraction verifies such relevance. These findings are highly relevant to government, industry, and environmental non-governmental organisations (including anti-shale-gas campaigners and groups interested in renewable energy, such as geothermal) as the UK Government continues its deliberation on future energy policy.

### Public perceptions of induced seismicity

Within the last decade, researchers have begun to explore the differences in how members of the general public perceive and respond to induced seismic events^38–43^. Risk research has long established that the public perceives voluntary and involuntary risks differently, accepting, for example, much higher risks associated with skiing than similar magnitude health risks from environmental exposure to toxic chemicals^44^. This understanding was extended further to reveal the notable difference in public acceptability of naturally occurring hazards when compared with human-induced hazards^45^.

In Oklahoma, for example, the substantial increase in induced seismicity caused by injection of wastewater from oil and gas operations has caused mental health concerns^46^, increased risk perceptions about shale gas development^38^, and has appreciably reduced trust in government regulators^39,42^. McComas and colleagues^41^ reveal that expert-driven processes around induced seismicity in New York (USA) are less acceptable to the public, compared to processes in which the public is afforded a role in deciding whether and how to implement the technology that caused the seismicity.

Evidence has emerged that public opposition to industrial processes leading to the seismic events (e.g., enhanced geothermal systems) can stop projects from moving forward, but also that outreach programs have been able to reduce opposition^47^. McComas and colleagues’^41^ experimental framing study, from the US, reveals not only that induced seismicity is perceived as more negative than the same seismicity from natural earth tremors, but also that when private companies benefit from the induced seismicity, this makes the seismicity less acceptable. Micro-seismic events can be caused by a large range of industrial processes, from surface and sub-surface mining to construction—anything that causes ground shaking. Vlek^48^ and Ritchie and colleagues^42^ point to the key role of trust in responsible experts and policy-makers, and perceived procedural fairness, in relation to induced seismicity from gas development in Groningen, The Netherlands, and wastewater injection in Oklahoma, USA, respectively. Liu and colleagues^49^ reveal that two distinct forms of trust are both relevant for public acceptability of gas development that may cause induced seismicity; perceived integrity of responsible actors is even more important than competence-based trust.

Although research is beginning to scratch the surface of how the public interacts with induced seismicity, risk scholars have made a convincing case for the need for more social scientific and risk communication inquiry into public understanding and reactions^43^. They argue that only after knowing an audience’s knowledge, associations, and needs can effective risk communication messages be produced. The way in which information is provided (e.g., numerical vs narrative^40^), along with the strength and valence of existing views, trust in the information source, institutional relationships, and other factors shape how risk information is perceived and used^50^. Novel approaches to risk communication are seen as essential^48^, especially considering that an individual’s own perceived knowledge insufficiency about induced seismicity has been shown to lead to risk information avoidance^51^. On the other hand, providing information does not always change risk perceptions or lead to policy support—indeed it may serve only to reinforce existing views and polarise opinion, particularly for contentious issues^52^.

## Research design

Much previous research on how the public thinks about and responds to earthquakes has sought to identify ways of communicating with the public in an effort to make them more risk-aware and to actively encourage them to take protective adaptations to reduce risk of harm^53–56^. As such, previous studies on earthquake risk perceptions have mostly focused on low-probability high-consequence events^40^. Our research explicitly takes up the opposite form of induced seismicity, that of high-probability low-consequence events (i.e., unlikely to cause surface damage). In particular, we use an experimental design to test for the first time whether giving people a range of information about seismicity influences their support for policy changes associated with shale gas extraction.

**Figure 1:**
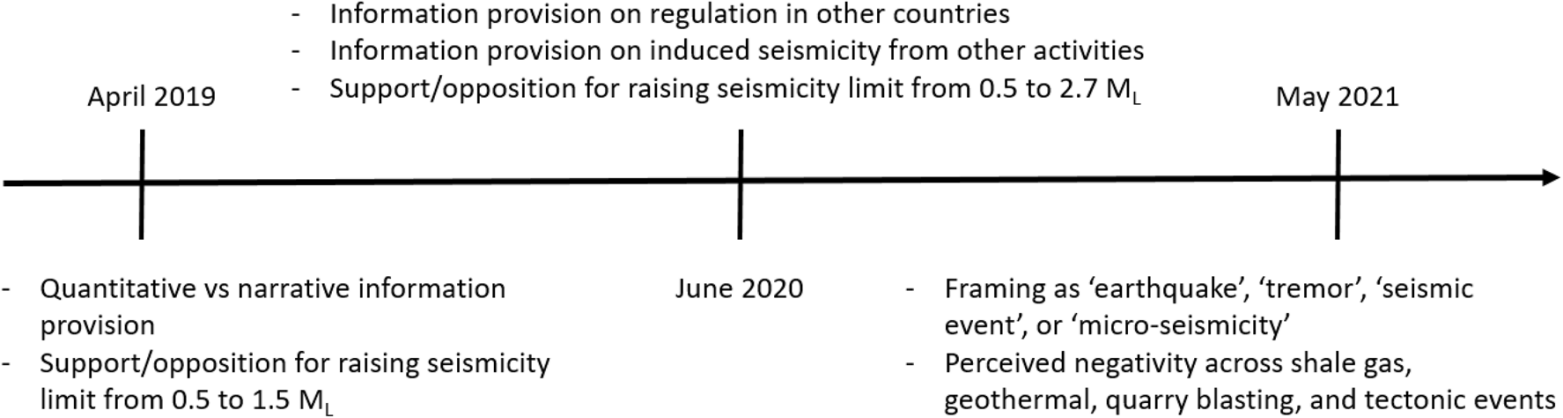
Three waves of surveys on perceptions of induced seismicity.

Our first survey builds on research showing that how technical or numerical risk information is conveyed makes a difference for how induced seismicity is understood and responded to^40,57,58^. We compared a quantitative description of the difference between 0.5 and 1.5 M_L_ with a narrative description in which we use analogy to convey the difference (see “Methods”). We sought to test narrative framing^59^ because the public may not adequately understand the complexity of logarithmic values, even though Knoblauch and colleagues^40^ showed in a Swiss sample that quantitative framing of seismicity risks was preferred. Figure 1 depicts our multi-stage investigations visually.

Our second wave of the longitudinal survey included experiments comparing support for changes in shale gas policy across conditions in which additional information was provided about regulations in other countries, and about effects of induced seismicity from other human activities. Rather than being informed based on theoretical or empirical literature, these wave 2 investigations arose from conversations with geoscience colleagues and members of the oil and gas industry we interacted with at professional meetings. When presenting our results from the first survey, they were incredulous that support for policy change was low and alleged that if people ‘only knew’ the additional information, they would naturally perform a *volte-face*.

In our third wave of the survey, we conducted another framing experiment, giving four different descriptions of ground movement—each to 25% of the sample: earthquake, tremor, micro-seismicity, and seismic event. We investigated differences in perceived negativity of the event, based on framing condition. For each respondent, we also examined their perceived negativity across different activities leading to the seismic event: shale gas extraction, deep geothermal operations, quarry blasting, and natural tectonic movements.

Our explicit research questions were:

1. To what extent do various framings (quantitative/qualitative, wording of seismic event) and information provision (on regulation in other countries, on relationship to other activities) affect support for policy change on shale gas extraction?
2. To what degree does the cause of the seismic event influence perceptions of induced seismicity?
3. What attitudes and beliefs most shape support for policy change (beyond effects of framing and information provision)?
4. What are the implications of the responses to the foregoing questions for communication about and policy on induced seismicity from energy development?

We initially surveyed a sample of 2,777 UK residents in April 2019—administered by the online panel provider YouGov and representative of the UK population based on age, sex, census region, social grade, education, vote in the 2017 general election, vote in the 2016 EU referendum, and attention paid to politics. This same sample was invited to a follow-up survey in June 2020, attracting 1,858 respondents (67% from wave 1). The respondents to wave 2 were invited to a third wave, run in May 2021, which had 1,439 respondents (52% from wave 1).

## Results

### Opposition to policy change

In wave 1, we provided explicit information in our survey that none of the tremors caused by hydraulic fracturing in the UK in 2018 caused damage, and that only two were strong enough to be felt by humans (see “Methods”). By wave 2, larger seismic events had occurred in association with shale gas development in the UK, and hydraulic fracturing had been halted. We then stated that ‘tremors, of 2.1 and 2.9 M_L_, exceeded the government’s allowed limit of 0.5 M_L_, and stopped operations temporarily’, and that this contributed to the English moratorium.

Our respondents provided a clear indication that they opposed changes to the UK induced seismicity limit. After reading the background information, wave 1 respondents, on average, slightly to moderately opposed an increase of allowable seismicity from 0.5 to 1.5 M_L_ (Table 1). Respondents also perceived the 0.5 M_L_ limit, on average, somewhere between ‘somewhat loose’ and ‘about right’. Thirty-one percent of the sample replied ‘don’t know’ to both of these questions. The leading predictor of ‘don’t know’ responses was respondents also indicating that they read or heard ‘nothing at all’ about ‘earth tremors linked to shale gas extraction’ (66% for ‘don’t know’ respondents, vs 31% for respondents who selected any other answer to the limit change; with 67% vs 31% for the stringency question).

**Table 1:** Wave 1 survey: policy support across information conditions.

	Control	Qualitative^d^	Quantitative^d^	Both
Traffic light system loose or stringent regulation?^a^	2.31^c^	2.29	2.43	2.44
Support/oppose limit change from 0.5 to 1.5 M_L_?^b^	2.59	2.68	2.69	2.83

^a^Five-point bipolar scale, with don’t know (DK = 31%) (1 = Far too loosely, 2 = Somewhat loosely, 3 = About right, 4 = Somewhat stringently, 5 = Far too stringently).

^b^Six-point bipolar scale, with don’t know (DK = 31%) (1 = Strongly oppose, 2 = Moderately oppose, 3 = Slightly oppose, 4 = Slightly support, 5 = Moderately support, 6 = Strongly support).

^c^Following ANOVA tests/, we ran Tukey post-hoc tests for differences between means of the two policy-relevant attitudes, across the four treatment conditions. Results revealed no significant differences.

^d^See methods for text of qualitative and quantitative information provision.

We asked about support/opposition towards a policy change of increasing the limit for seismicity from 0.5 to 1.5 M_L_ in our April 2019 (wave 1) survey, because this had been the explicit recommendation of numerous geoscientists in open letters in early 2019^4,60^. However, following the larger seismic events in August 2019 (2.1 and 2.9 M_L_), we then asked about support for policy change increasing the limit from 0.5 to 2.7 in our June 2020 (wave 2) survey. We stated that 2.7 is ‘the limit used in Switzerland and California (USA)’^61^. The wave 2 respondents, on average, slightly opposed the limit increase (Table 2). Likewise, they slightly opposed removing the moratorium on shale gas extraction that arose, at least in part, due to the seismic events.

**Table 2:** Wave 2 survey: policy support across information conditions.

	Control	Other limits^b^	Other activities^b^
Support/oppose removing the moratorium on shale gas extraction?^a^	2.96^c^	3.06	3.08
Support/oppose limit increase from 0.5 to 2.7 M_L_?^a^	3.13	3.30	3.29

^a^Six-point bipolar scale, with don’t know (DK = 16% for removing the moratorium, 20% for increasing the limit) (1 = Strongly oppose, 2 = Moderately oppose, 3 = Slightly oppose, 4 = Slightly support, 5 = Moderately support, 6 = Strongly support).

^b^See methods for text of information provision on ‘other limits’ and ‘other activities’.

^c^Following ANOVA tests, Tukey post-hoc tests for differences between means of the two policy-relevant attitudes, across the three treatment conditions, revealed no significant differences.

### Effects of information provision and framing

The lack of significant differences in any of the ANOVA post-hoc tests in Tables 1 and 2 suggests that ‘information deficit’ explanations for opposition to policy changes surrounding induced seismicity lack credibility and nuance^62^. Providing additional qualitative information, quantitative information, or both forms of information, led to no differences from the control group that received none of this information in wave 1 (see “Methods”). Likewise in wave 2, additional information on regulatory limits that are far higher in jurisdictions other than the UK, and information about other activities that lead to similar ground shaking as induced seismicity from shale gas, led to no difference in support for policy change from the control group that did not receive any of that information (see “Methods”). This raises the question of which factors did influence support for, or opposition to, policy change on induced seismicity.

### Why was policy change opposed?

Various factors affected support for changing the traffic light limit in survey waves 1 and 2 (Table 3). Clearly, the two most important predictors in both models are the response to how negative respondents would perceive an earthquake that could be felt but that caused no damage, and beliefs about procedural justice (the extent to which the public needs a voice in decisions on energy projects)—higher negativity and higher importance of public voice increase opposition. In both models, beliefs about the likelihood of tremors causing damage at the surface were also important predictors of opposition, and self-reported knowledge of induced seismicity was irrelevant (non-significant; wave 1) or a very minor influence (wave 2).

**Table 3:** Factors predicting support for changing the limit of seismicity allowed for shale gas extraction (linear regressions).

	0.5 to 1.5 M_L_ (wave 1) R^2^ = 0.31	0.5 to 2.7 M_L_ (wave) R^2^ = 0.63
‘How negative would you feel about an earthquake in your local area caused by shale gas extraction, which you could feel but that caused no damage?’	− 0.31**	− 0.49**
Likelihood that tremors will cause damage at the surface	− 0.08**	− 0.12**
How much read/heard about earth tremors linked to shale gas extraction	− 0.03	** − 0.04**
Objective numeracy (number correct of three items)	− 0.04	− 0.07**
Subjective numeracy (mean of three items)	0.00	**0.06**
Trust in industry groups or firms	0.11**	0.12**
‘Extraction is likely to have a big impact on people like me’	− 0.10**	** − 0.04**
‘The public needs to have a voice in decisions such as approving or refusing an application for a shale gas well.’	− 0.14**	− 0.17**
Perceived seriousness of climate change (mean of 4 items)	− 0.03	–0.11**

NB: Numbers in the cells are standardised beta coefficients. A positive coefficient indicates the variable associates with increased support for the policy change. Bold coefficients are statistically significant at *p* < 0.05. With one asterisk (*), *p* < 0.01; with two asterisks (**), *p* < 0.001. Independent variables measuring objective numeracy, subjective numeracy, and need for public voice come from wave 1 (April 2019) for both regressions; the other six independent variables were measured in both surveys.

Because seismicity measurement is a mathematical concept, we included measures of ‘numeracy’. We operationalised numeracy—literacy with numbers—according to established scales for objective numeracy (how good people are with numbers) and subjective numeracy (how good people think they are with numbers)^63–65^. In wave 2, respondents who thought they were good with numbers supported policy change, whereas those who were objectively good with numbers were more opposed to policy change.

An additional change from wave 1 to wave 2 is that perceived seriousness of climate change (an average of perceived seriousness to: you and your family, the UK as a whole, people in developing nations, and wildlife and ecosystems) became significant in wave 2. This may relate to the increased public attention to climate change between April 2019 and June 2020. The overall model effect size (R^2^—the percentage variance in the dependent variable explained by the group of independent variables) doubled from 31% to 63% from wave 1 to wave 2.

Arguably, there is very low probability of any structural damage at the surface from induced seismicity due to shale gas extraction^6,8^; nevertheless, 59% responded that this is somewhat likely or very likely in wave 1, and 68% so responded in wave 2. This belief was significant in both regressions (Table 3)—more perceived likelihood of damage increases opposition to policy change. Nevertheless, perceived negativity of an earthquake that *causes no damage* was the most predictive variable of opposition to policy change in both models. Therefore, it is not merely misconceptions about the effects of induced seismicity that shapes public views—those misconceptions do influence the model results, but to a lesser extent, particularly in wave 2 when the importance of perceived negativity of non-damaging earth tremors and climate change severity increased in their predictive capacity (see beta coefficients in Table 3).

**Figure 2:**
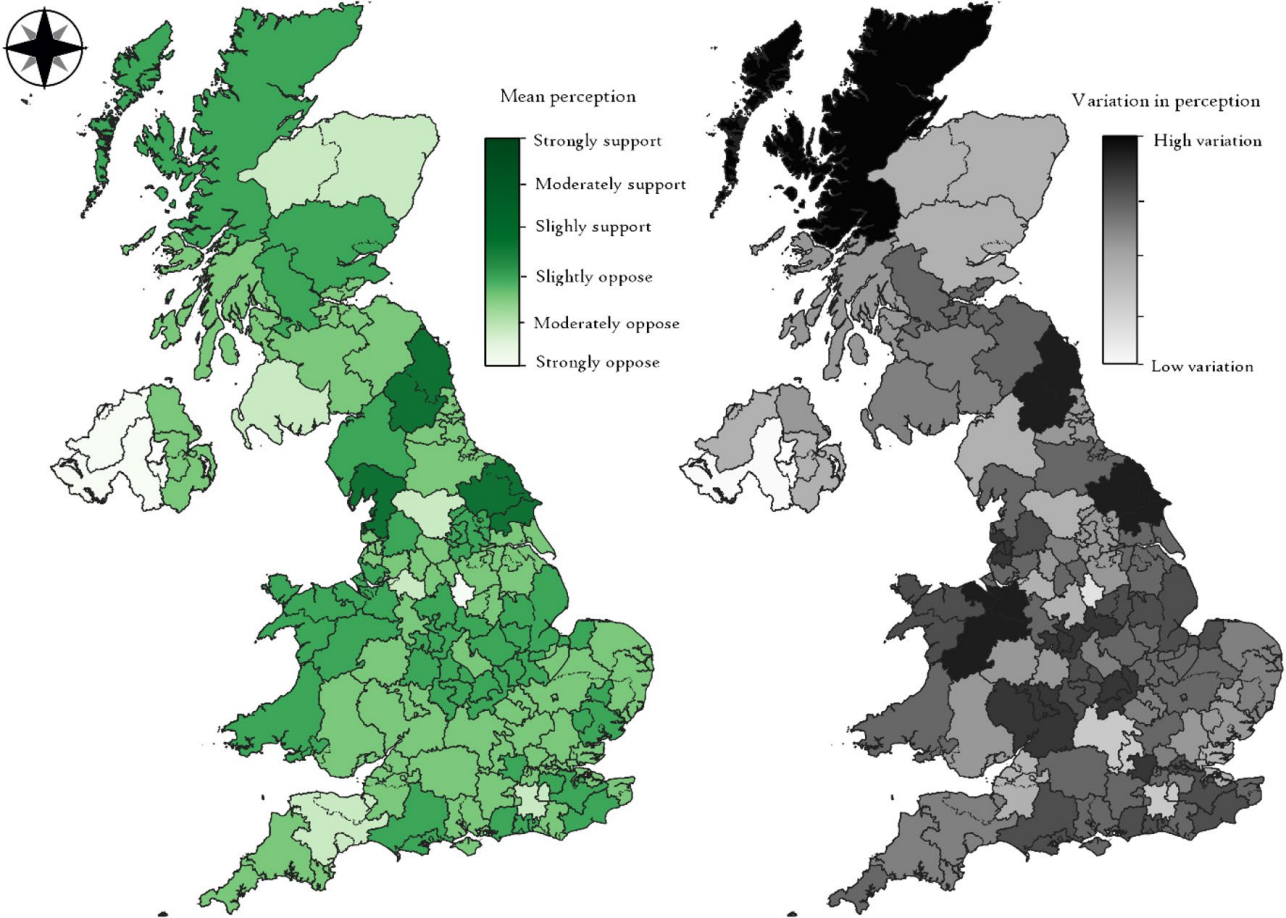
Spatial distribution of support or opposition for changing the traffic light limit from 0.5 to 1.5 M_L_ (wave 1).

A further factor that might influence support for policy change is geospatial distribution of respondents. Although UK census region (12 broad regions covering the UK) was not a significant predictor in the regressions, and other means of aggregating spatial areas (e.g., local authority level) did not offer sufficient sample sizes for determining statistical significance, a visual representation of survey responses across political constituencies (with some aggregated) reveals patterns. Figure 2 (see “Methods” for data aggregation) displays a general pattern of stronger support for policy change in the north of England, compared to the south, which is more opposed. In April 2019, there were far more prospects for and discussion of shale gas extraction happening in Northern England than in Southern England. Nevertheless, no region represented has a maximum value over 3.8 on the six-point scale; therefore, no values even reach the level of ‘slightly support’. The graphic on the right in Fig. 2 reveals that areas with generally more support are also more divided/polarised on policy change (higher standard deviations).

### Differences across activities generating induced seismicity

The wave 1 and wave 2 surveys offer an understanding of attitudes and beliefs that shape support/opposition towards policy changes relevant to induced seismicity, and convincingly demonstrate very little role for framing or information provision in influencing such support/opposition. In our wave 3 survey, we explored one final framing condition—if the way the event was described affected perceived negativity to induced seismicity, and if that perceived negativity varied across causes of events.

Some small differences emerged for the framing test. For the questions about seismicity from ‘deep geothermal operations’ or ‘quarry blasting’, induced seismicity was viewed as more negative when framed as an ‘earthquake’, as opposed to ‘micro-seismicity’ or a ‘tremor’ (Table 4). Nevertheless, for ‘shale gas operations’ and ‘natural movements of the earth’s tectonic plates’, no significant differences existed across any of the four framing conditions. Furthermore, the significant differences only became manifest when the geothermal and quarry blasting events were described as being felt; for unfelt events, framing condition was non-significant across all four activities leading to seismicity.

**Table 4:** Wave 3 survey: perceived negativity of seismicity of events, based on framing and cause of the event.

How negative would you feel ______ is, caused by…	‘An earthquake’	‘A seismic event’	‘A tremor’	‘Micro-seismicity’
Shale gas extraction (can feel ground movement, no damage)^1^	7.82	7.55	7.56	7.36
Deep geothermal operations (can feel, no damage)	7.21^a 2^	6.74^a,b^	6.63^b^	6.54^b^
Quarry blasting or similar industrial processes (can feel, no damage)	7.41^a^	7.04^a,b^	6.84^b^	6.79^b^
Natural movements (can feel, no damage)	5.15	5.13	5.02	4.80
Shale gas extraction (cannot feel ground movement, no damage)	6.34	6.09	5.93	5.98
Deep geothermal operations (cannot feel, no damage)	5.76	5.15	5.10	5.10
Quarry blasting or similar industrial processes (cannot feel, no damage)	6.08	5.67	5.45	5.51
Natural movements (cannot feel, no damage)	4.19	3.99	3.94	3.72

^1^All items in this table were measured on an eleven-point scale, 0 = not at all negative, 10 = very negative.

^2^Within a given row, superscript letters denote values that differ from each other significantly, based on Tukey post-hoc tests for differences between means, following an ANOVA. If the letter is the same, those mean values do not differ. If a row has no superscript letters, there are no significant differences amongst the four means for that item.

Of all our comparisons, we observed by far the largest differences in the perceived negativity of the event when comparing across different types of activities leading to induced seismicity. On average (combining all framing conditions), hypothetical events from shale gas operations that one could feel but that did not cause damage were rated as 7.55 on a scale of 0 (not at all negative) to 10 (very negative). The exact same description of the event, but caused by ‘quarry blasting or similar industrial processes’ was rated 7.05; for deep geothermal operations, it was 6.80. All respondents were presented with questions on perceived negativity for the three activities, and also natural tectonic movements—mean of 5.08.

A repeated-measures ANOVA test, comparing views on the four different causes of seismicity by the same respondent, had a very large eta^2^ (effect size) value of 0.26. If removing the outlier of the natural tectonic movements, the eta^2^ was still moderately strong at 0.07—showing that the exact same seismic event is viewed significantly more negatively when arising from shale gas operations as opposed to being from other causes, both anthropogenic and natural. Geothermal-induced events were notably less negative than shale gas operations and quarry blasting (all four items differed significantly at *p* < 0.001), but their mean perceived negativity was also clearly closer to the other induced events than to the natural tectonic movements (Table 4).

## Discussion

### Implications for policy and communication

The core messages from our data are clear: the *cause* of a seismic event is a strong influence on perceived negativity of the event, and perceived negativity is then a leading predictor of support/opposition towards policy change. The explanatory power of this predictor notably increased over time from our wave 1 survey to wave 2. Furthermore, there is little support for any policy change in relation to regulation of induced seismicity for shale gas extraction in the UK.

Perhaps the most interesting question arising from the research reported herein is what our findings presage for activities such as deep geothermal operations. Although perceived negativity of geothermal-induced seismicity was significantly lower than for shale gas-induced seismicity, and with moderate effect size, geothermal seismicity was still seen as substantially more negative than natural tectonic movements leading to the same event. Although we are not aware of any research on perceptions of induced seismicity in relation to compressed air storage, sub-surface hydrogen storage, or carbon capture and storage, one might speculate at best similar negativity to geothermal, especially considering likely less perceived benefit from less ‘green’ technologies. Research in Switzerland revealed induced seismicity risk perceptions as the leading variable predicting (lack of) acceptance of deep geothermal energy^66^.

*Policy change*, nevertheless, may not be necessary for deep geothermal operations to advance in the UK. Regulation of geothermal seismicity occurs at the local authority level in the UK (as opposed to the national level) and uses peak ground velocity (PGV) during an earthquake as the measure for the ground shaking. PGV correlates well with seismic intensity^67^ and damage from the small, shallow earthquakes caused by geothermal operations^68–70^. In Cornwall, where the March 2022 geothermal-induced 1.7 M_L_ event occurred, the maximum PGV permitted is 8.5 mm per second^71^. In contrast, the 2.9 M_L_ event in August 2019, the highest ever UK tremor during the shale gas operations, recorded a PGV of about 8.2 mm/s^72,73^—below the limit set for geothermal operations in Cornwall. This suggests legitimate potential for deep geothermal to move forward. Less hydraulic fracturing is needed for enhanced deep geothermal than for shale gas, and no policy change seems required.

For people interested in the future of geothermal as a renewable energy industry in the UK, the lack of a need for policy change is quite advantageous—considering that our repeated efforts at finding any framing conditions or information provision that could influence support for policy change came up mostly empty-handed. Across the framing conditions and information presented in the three surveys, the only (minor) effect was seen in the language of ‘earthquake’ leading to slightly more negative evaluations, compared to ‘micro-seismicity’ and ‘tremor’ (but ‘seismic event’ did not differ from any of the other three).

As demonstrated by McComas and colleagues^41^, tolerance for any induced seismicity (i.e., a change in natural processes) can be quite low if few perceived benefits to society are seen as accruing from the practice, and it is being conducted by a corporate actor with little trust. Twelve percent in our survey (wave 1) reported trusting the shale gas industry ‘a fair amount’ or ‘a great deal’, whilst 40% ‘do not trust at all’ industry actors (9% trust and 46% no trust for wave 2). No clear data exist on trust in the UK geothermal industry, but we would hypothesise that public trust is considerably higher than in the shale gas industry, and that perceived benefits from, and general positivity towards, geothermal development are also higher^74–76^. Future research could explore in greater detail what the public know about deep geothermal, their associations with seismicity, their trust in key actors, and how these affect acceptance of this form of energy development.

### Future of shale gas in the UK

Despite the aforementioned industry calls for reconsideration of shale gas extraction, we do not foresee a role for shale gas in the UK’s energy future. Bradshaw^14^ convincingly explains that the timeline to commercial production is too long for shale gas to help with supply issues in the short term; in the long term, expanded gas production conflicts too notably with net-zero targets. Furthermore, initial indications suggest that even with Russian supply concerns, MPs (including Conservatives) in areas with shale gas potential are not coming out in favour of renewed exploration^77^. Any attempts to change the policy landscape to make hydraulic fracturing viable by increasing the seismicity limit would likely be met with stark resistance. Protests in relation to shale gas extraction are common in the UK, and discourse about UK decision making on shale gas suffering from democratic deficits has been increasingly common^11,78–81^. Furthermore, climate change protests and demonstrations have substantially expanded in the UK since 2019, and shale gas is tied increasingly to its implications for global carbon emissions. The role of climate change beliefs in influencing policy support became important in our wave 2 survey.

One might argue that communication efforts to influence public opinion could be undertaken in advance of any policy change. Leaving aside that such communication seeking to change attitudes is extremely difficult^82^ and that attitudes towards shale gas extraction have only shifted slightly over ten years^18,83^, our data reveal further reasons to question the success of such efforts. Framing and information provision had little to no effect on support for policy change or perceived negativity of induced seismicity. One explanation for lack of effect of communication on attitudes could be that attitudes are well-established already, not only from exposure to news on shale gas extraction, but in some areas of the UK perhaps due to exposure to induced seismicity from historical processes such as coal mining (last seismic event in 2014) and quarry blasting.

Perceived negativity of hypothetical non-damaging seismic events was the lead predictor of support/opposition towards policy change. People in the UK are opposed to the *existence* of the seismic events, not primarily to their effects. This is consonant with McComas and colleagues^41^, showing lack of support for ‘unnatural’ events especially if there is little perceived social benefit, and Ritchie and colleagues^42^, showing the connections between withdrawn trust and opposition to industrial operations. Research further shows that factual beliefs about shale gas development may stem from negative attitudes towards shale gas, rather than the beliefs fostering such attitudes^84^. Even if factual beliefs about prospects for damage from induced seismic events could be changed^85^, it would not likely translate to meaningful shifts in support for new policies.

## Methods

### UK context

From mid-October 2018 through January 2019, hydraulic fracturing at a single shale gas well in Lancashire, England, led to detection of over 60 micro-seismic events, registering from -0.8 to 1.5 M_L_, with two of these reported as being felt by a few members of the local public (1.1 and 1.5 M_L_)^6,8,72^. The UK Oil and Gas Authority (OGA; now called the North Sea Transition Authority) was established in 2015 as the regulator for the UK oil and gas industry. It adopted a ‘traffic light system’, which requires that if a seismic event of 0.5 M_L_ or higher occurs in the vicinity of a gas well during hydraulic fracturing^86^, the operator ‘must immediately suspend injection, reduce pressure and monitor seismicity for further events’^87^. This threshold has been exceeded multiple times, leading to suspension of operations for at least 18 hours in each instance.

Controversy arose in the UK in 2018–2019 over whether the low threshold for suspension of operations under the traffic light system should be raised or not. Leading seismologists from the British Geological Survey also entered the debate, noting that 0.5 M_L_ is a ‘really quite conservative’ threshold and lower than required to prevent harm to humans or built structures^60^. They contended that 1.5 M_L_ would still be conservative. Repeated articles in major national news media outlets covered the debate over the traffic light system as well as occurrences of several individual micro-seismic events. It was in this context of societal attention to hydraulic-fracturing-induced seismicity that we began our research.

### Survey design, implementation, and analysis

We expected few survey respondents to have any background on induced seismicity in association with shale gas extraction. In the wave 1 survey, after three initial questions about perceptions of the likelihood of tremors due to shale gas extraction, self-assessed knowledge on the topic, and level of negativity towards hypothetical tremors, we provided all respondents with the following two paragraphs of information:

Recent observations by the British Geological Survey (BGS) have linked ‘induced micro-seismicity’ (small earth tremors that are mostly not felt by humans) to shale gas extraction. About sixty such tremors occurred at a shale gas extraction site in Lancashire between October and December 2018. Only two tremors were strong enough to be felt by humans; none caused damage at the surface.

The UK’s Oil and Gas Authority has a ‘traffic light system’ in place that requires hydraulic fracturing to be paused for 18 hours if a tremor of 0.5 magnitude or higher is recorded. This happened several times at the well site. Some scientists have recently written an open letter stating their view that the magnitude at which fracturing is paused can safely be raised from 0.5 to 1.5.

In addition to information provided to all respondents, sub-samples received further information about induced seismicity. We provided different messages to random samples of the respondents taking our survey (25% of the sample in each condition). In the first condition, we provided only the general background above. In the second condition, we used the same language, save adding a quantitative explanation of M_L_:

Because seismic magnitude is measured logarithmically, the largest tremor in 2018 (1.5 magnitude) is 316 times smaller than the level at which a tremor would cause structural damage at the surface (4.0 magnitude).

In the third condition, we added to the background qualitative descriptions of events to which a 1.5 M_L_ event is similar (generated through conversation with seismologists at the BGS):

A 1.5 magnitude tremor occurring 1 km underground is similar to the vibrations felt in a home with a concrete floor when a heavy goods vehicle (HGV) travels down a road 200 feet from the house.

In the fourth condition, we included the additional wording of both the second and third conditions.

In the wave 2 survey (June 2020), after responding again to the initial questions about perceived likelihood of damage from tremors, self-reported knowledge of earth tremors due to shale gas development, and negativity of induced seismicity, we provided all respondents with the following text:

In August 2019, two seismic tremors occurred at a shale gas well in Lancashire, due to use of hydraulic fracturing. These tremors, of 2.1 and 2.9 magnitude, exceeded the government’s allowed limit of 0.5 magnitude, and stopped operations temporarily. In November 2019, the UK Government placed a moratorium (temporary ban) on use of hydraulic fracturing in England due in part to questions over whether seismic tremors can be appropriately managed.

Our message testing experiment then examined whether provision of additional further information would affect support for policy changes. The first condition included the following further text:

Prior to the moratorium, the English threshold of limiting allowed seismicity from hydraulic fracturing to 0.5 magnitude was more stringent than in other regions. For example, the limit is set at 2.7 in both California (USA) and Switzerland, and 4.0 in Alberta and British Columbia (Canada) and in Illinois (USA).

The second condition added the following text to the wave 2 background:

The largest seismic tremors from the August 2019 hydraulic fracturing were felt by a few individuals local to the well site. The ground vibrations associated with these tremors are similar to those experienced on average a few times annually in the UK from other types of industrial activity, such as coal mining and quarry blasting.

The third condition, the control, included only the initial text.

The third survey wave (May 2021) asked a series of eight questions to each respondent about perceived negativity of induced seismicity. The first four questions asked about events ‘in your local areas’ that ‘you could feel’, but that ‘caused no damage’. The four questions were identical, except that the cause of the event changed in each:

- Shale gas extraction
- Deep geothermal operations (drawing renewable heat from rocks far underground)
- Quarry blasting or similar industrial processes that occur above ground
- Natural movements of the earth’s tectonic plates

Additionally, the event was described differently for different respondents. Twenty-five percent of the sample was randomly assigned to each condition: ‘an earthquake’, ‘a tremor’, ‘micro-seismicity’, and ‘a seismic event’. The second four questions were the same as the first, with the framing of the event consistent for each respondent. The only difference was that the event was explicitly described as ‘minor’, and still did not cause damage, but now could *not* be felt.

For data analysis of the survey results, we employed analysis of variance (ANOVA) tests with post-hoc comparisons to compare means on support for policy changes in wave 1 and wave 2. The policy support variables were treated as linear, which meant excluding ‘don’t know’ responses as missing data. The factor variables in the ANOVAs were the aforementioned information provision categories. We then conducted linear regressions to assess relative influence of various attitudes and beliefs on support/opposition towards changing the induced seismicity limit. For the wave 3 survey data, ANOVAs were used to examine for differences in mean perceived negativity across the framing conditions within each of the eight negativity questions. Then a repeated measures ANOVA was used for examining within-subjects differences across each given individual’s responses to the four different causes of seismic events.

Human subjects approval for the survey research was granted by the Ethics Committees of the School of Social and Political Sciences at the University of Edinburgh and the Geography department at the University of Exeter. Informed consent was obtained from all research participants. All methods were performed in accordance with the relevant guidelines and regulations.

### Spatial analysis

**Figure 3:**
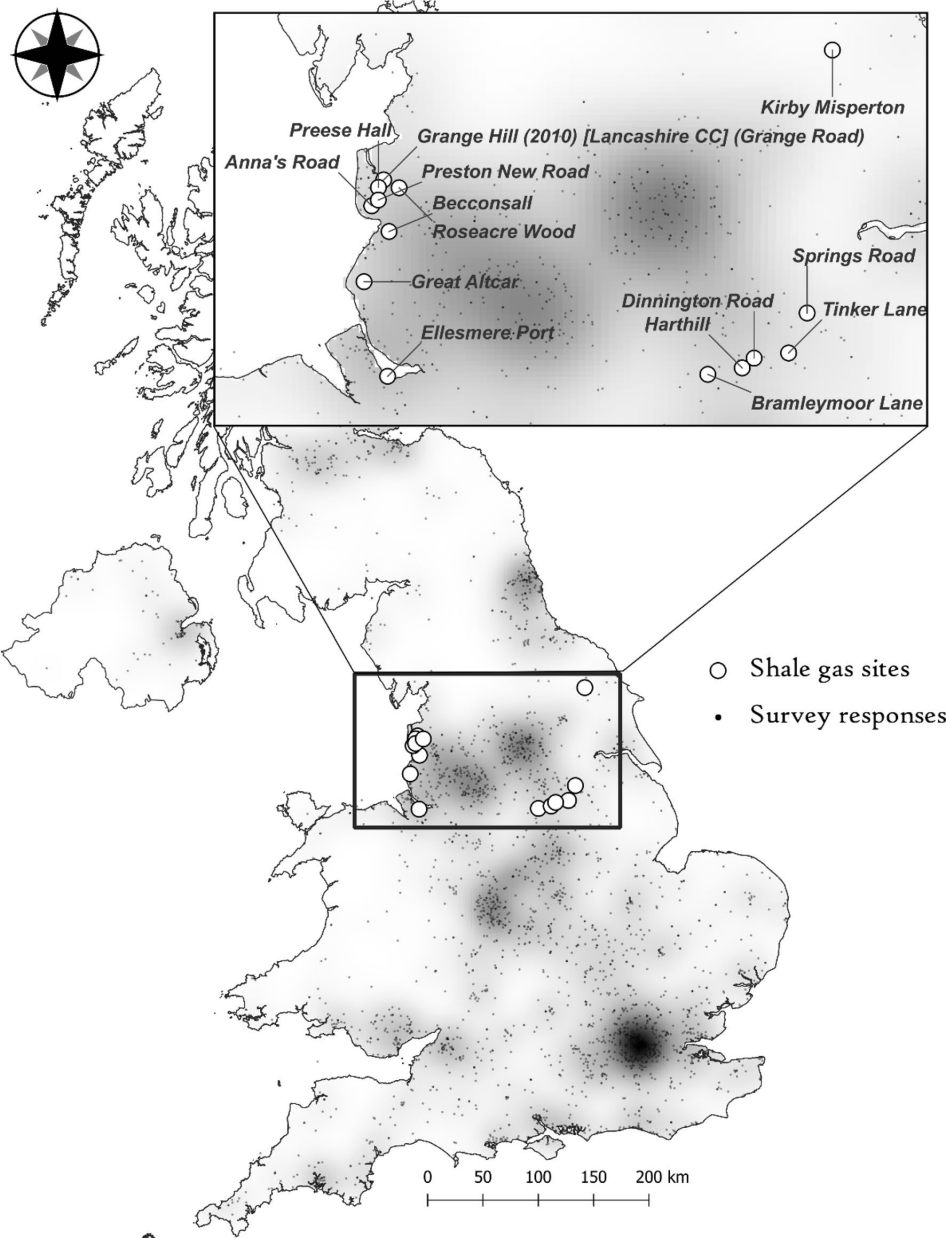
Spatial distribution of survey respondents plotted alongside sites that have been associated with shale extraction.

For the vast majority of respondents, YouGov provided the first four digits of their postcode, allowing individual responses to be georeferenced to approximately a kilometre accuracy using UK postcodes^88^. The distribution of respondents across the UK alongside shale-associated sites is displayed in Fig. 3.

Raw data were too sparse to map by themselves, or for spatial interpolation algorithms, therefore respondents were aggregated using polygon boundaries defining political constituencies. Within the UK, there are 650 constituencies that are for the most part conveniently defined around population density. Nonetheless, for some parts of the survey area constituency boundaries were too small to provide enough statistical accuracy to present a clear spatial pattern across the UK. Higher statistical certainty was achieved by dissolving some of the constituencies, notably those in rural areas in Scotland and Wales into larger regions using a nearest neighbour algorithm to obtain a minimum of 15 respondents within each polygon. Naturally, this process lead to a degree of spatial resolution loss in these areas, but we deemed it necessary to gain a more robust insight into spatial patterns. After applying the algorithm, 151 polygons were used to calculate the mean and standard deviation for all respondents contained within bounding polygons.

## Data Availability

The datasets used and analysed during the current study are available from the corresponding author upon reasonable request.
